# Chronic kidney disease of unknown origin is associated with environmental urbanisation in Belfast, UK

**DOI:** 10.1007/s10653-020-00618-y

**Published:** 2020-06-24

**Authors:** Jennifer M. McKinley, Ute Mueller, Peter M. Atkinson, Ulrich Ofterdinger, Siobhan F. Cox, Rory Doherty, Damian Fogarty, J. J. Egozcue, V. Pawlowsky-Glahn

**Affiliations:** 1 School of Natural and Built Environment, Queen’s University Belfast, Belfast, Northern Ireland; 2 School of Science, Edith Cowan University, Perth, WA Australia; 3 Lancaster Environment Centre, Lancaster University, Lancaster, UK; 4 Belfast Health Trust, Belfast, Northern Ireland; 5 Department of Civil and Environmental Engineering, U. Politécnica de Cataluña (UPC), Barcelona, Spain; 6 Department of Computer Sciences, Applied Mathematics, and Statistics, University of Girona, Girona, Spain

**Keywords:** Renal disease, Uncertain aetiology, Compositional data analysis, Soil geochemistry, Social deprivation measures, Tweedie model

## Abstract

Chronic kidney disease (CKD), a collective term for many causes of progressive renal failure, is increasing worldwide due to ageing, obesity and diabetes. However, these factors cannot explain the many environmental clusters of renal disease that are known to occur globally. This study uses data from the UK Renal Registry (UKRR) including CKD of uncertain aetiology (CKDu) to investigate environmental factors in Belfast, UK. Urbanisation has been reported to have an increasing impact on soils. Using an urban soil geochemistry database of elemental concentrations of potentially toxic elements (PTEs), we investigated the association of the standardised incidence rates (SIRs) of both CKD and CKD of uncertain aetiology (CKDu) with environmental factors (PTEs), controlling for social deprivation. A compositional data analysis approach was used through balances (a special class of log contrasts) to identify elemental balances associated with CKDu. A statistically significant relationship was observed between CKD with the social deprivation measures of employment, income and education (significance levels of 0.001, 0.01 and 0.001, respectively), which have been used as a proxy for socio-economic factors such as smoking. Using three alternative regression methods (linear, generalised linear and Tweedie models), the elemental balances of Cr/Ni and As/Mo were found to produce the largest correlation with CKDu. Geogenic and atmospheric pollution deposition, traffic and brake wear emissions have been cited as sources for these PTEs which have been linked to kidney damage. This research, thus, sheds light on the increasing global burden of CKD and, in particular, the environmental and anthropogenic factors that may be linked to CKDu, particularly environmental PTEs linked to urbanisation.

**Electronic supplementary material:**

The online version of this article (10.1007/s10653-020-00618-y) contains supplementary material, which is available to authorized users.

## Introduction

Chronic kidney disease (CKD) is a collective term for many causes of progressive renal failure. While CKD is increasing worldwide due to ageing and a general increase in obesity and diabetes, it is acknowledged that these key factors cannot explain the occurrence of environmental clusters of CKD of unknown causes. This has resulted in the establishment of a high-level task force convened by the WHO (WHO 2016) to explore the potential underlying environmental causes of CKD attributed to unknown aetiology (CKDu). CKD is associated with a natural decline in renal function over time, with a more rapid decline and resultant impact on life expectancy for individuals who have end-stage kidney disease (ESKD) (Lindeman et al. 1985; Musso and Oreopoulos 2011; McCrink and Marshall 2013). The United Kingdom Renal Registry (UKRR) collects case data regularly for all patients with advanced CKD on dialysis or with a kidney transplant (renal replacement therapy (RRT)) across the UK. These data are collected through several centres and include the diagnosis category “uncertain aetiology”. Since 2000, average annual prevalence of RRT increased by ~ 3.5% in the UK (Gilg et al. 2012), while from 2012 to 2013 prevalence of RRT grew by 4% (UK Renal Registry (UKRR) 2019). The incidence of RRT per million population (pmp) in 2017 was 121 pmp compared to 118 pmp in 2016 (UK Renal Registry (UKRR) 2019) 21st Annual Report—data to 31st December 2017). The UKRR (2019) reported that 8001 adult patients started RRT for ESKD in the UK in 2017, representing an increase of 2.6% from 2016 (Table 1). This trend is estimated to continue to increase for at least 25 years (Roderick et al. 2004). Increasing prevalence of CKD in the population indicates increasing demand on the health care system. In the UK, it is estimated that dialysis and transplantation (RRT) cost between £20,000 and £30,000 per patient per year (Lewis 2012).

**Table 1:** Summary statistics collected by the UK Renal Registry (UKRR) for number of patients with advanced chronic kidney disease (CKD) starting dialysis or kidney transplant therapy (renal replacement therapy (RRT)). Summarised from UKRR 2019

Centre N on RRT	2013	2014	2015	2016	2017	Est catchment population (millions)	2017 crude rate (pmp)
UK	7020	7457	7869	7797	8001	66.04	121
England	5978	6364	6619	6624	6771	55.62	122
Scotland	503	530	623	561	635	5.42	117
Wales	356	384	397	382	282	3.13	122
N Ireland	183	179	230	230	213	1.87	114
Belfast	72	65	94	96	75	0.66	114

Globally, while diabetes and hypertension are recognised as the predominant risk factors for CKD (Couser et al. 2011), environmental clusters of CKD have been reported in countries such as Sri Lanka, Central and Latin American countries, and regions within Egypt and India (Jha et al. 2013; Correa-Rotter et al. 2014; Weaver 2015; Gonzalez-Quiroz et al. 2018; GBD 2020). Endemic forms of CKD such as Mesoamerican nephropathies, Balkan endemic nephropathy and Chinese herbal nephropathy have been well documented (e.g. as discussed in reviews by Soderland et al. 2010; Weaver 2015; Afsar et al. 2019). In regional studies, CKDu appears to disproportionally affect poor, rural and, more frequently, male farmers living in hot climates. Risk factors have been linked to altitude and occupation resulting in increased exposure to nephrotoxins, oxidation stress and dehydration (Weaver 2015; Afsar et al. 2019). Research indicates that CKDu as a disease is more related to longer lifespans which provides time for CKD to develop and progress. In countries such as Sri Lanka and Central and Latin America, CKDu was found to affect adults in their third to fifth decade which often leads to fatal consequences due to disease progression and lack of dialysis or transplant options in the involved geographic areas (Weaver 2015).

Lead (Pb), cadmium (Cd), mercury (Hg) and arsenic (As) have been reported as environmental nephrotoxins (Soderland et al. 2010). Well-documented examples include the incidence of lead poisoning of children in Queensland, Australia, who survived but went on to die of end-stage kidney disease (ESRD) as adults. The exposure was linked to lead paint on painted verandas and railings of raised houses, a type of housing which was unique to Queensland until a ban in 1890 (cited in Weaver (2015) with original references in Henderson 1954, 1955; Henderson and Inglis 1957; Inglis et al. 1987). Excessive Cd exposure has been reported in several areas in Japan and attributed as the cause of Itai-itai (“ouch–ouch”) disease in the Jinzu River basin of Toyama prefecture (Weaver 2015). High levels of exposure to Cd and As leading to an increased risk for incident CKD have occurred through the ingestion of rice irrigated with Cd-rich industrially polluted water (Nogawa et al. 1980; Nogawa and Kido 1993) and elevated As levels in water sources (Zheng et al. 2014; in Taiwan, Chiu and Yang 2005; Zheng et al. 2015; in Sri Lanka, Jayatilake et al. 2013; Jayasumana et al. 2014). A recent review of the impact of air pollution on kidney damage outlined the scientific evidence that air pollution harms the kidney, and the detrimental effects of air pollutants, including toxic metals (Cd, Pb, uranium (U)), traffic and smoking (Afsar et al. 2019). As, molybdenum (Mo), tin (Sn), antimony (Sb) and Pb have all been linked to atmospheric pollution deposition including traffic pollution (Carrero et al. 2013). Brake lining and brake wear emissions have also been shown to be potentially important sources of iron (Fe), copper (Cu), zinc (Zn), Pb, Sb and Mo (review by Grigoratos and Martini 2015). The significance for investigating explanatory factors for CKDu is that studies have shown that ultrafine particles of these environmental toxins (including Pb, Mo and Sb) may become blood-borne and translocate to other tissues such as the liver, brain and kidney (Geiser 1999; Oberdörster et al. 2005).

Despite more than 20 years of study, CKDu is not well understood and the World Health Organisation (WHO) states that there remains no global definition for this disease (Jayatilake et al. 2013). Weaver (2015) concludes that none of the previously established causes alone appear to explain CKDu and that the disease may be multifactorial, possibly including explanatory factors such as ethnic diversity, late diagnosis due to limited access to health care and lack of diagnostic capability in some of the regional case studies. However, the environmental factors that may cause CKDu require further exploration as CKDu has been reported in individuals without the known risk causes, and therefore environmental factors may also be relevant to the heterogeneity of progressive CKD in diabetes and hypertension.

Using an urban soil geochemistry database of total element concentrations, we explored the statistical relationship between standardised incidence rates (SIRs) of CKD and CKDu and both social deprivation measures and environmental factors.

### Data and study site

Belfast urban area, the capital of Northern Ireland (NI), UK, was used as the study site for this research. The Belfast City Council reports that Belfast has a population of 340,220 in the city and over one million people in the greater Belfast region (Belfast City Council 2019).

### CKD classification

In addition to demographics, the UKRR collects data on all patients with advanced CKD on dialysis or with a kidney transplant (RRT) across the UK and reports data by age group on primary renal disease. For the 71 centres across the UK, the number of incident patients on RRT is calculated as a proportion of the estimated centre population. For this research, the UKRR provided SIRs for patients starting RRT between 2006 and 2016, by Super Output Areas (SOAs) which are the smallest administrative wards in Northern Ireland. There are 890 SOA administrative wards across Northern Ireland. Data were provided in age brackets, 16–39, 40–64 and 65 + , all ages > 16 and for uncertain aetiology (CKDu) between 2006 and 2016. The SIR for a SOA is a measure that quantifies the relationship between actual incidence in the SOA and the expected incidence based on that of Northern Ireland as a whole. SIRs of exactly 1 indicate that a SOA’s incidence for RRT is equal to that expected based on Northern Ireland’s average age-specific incidence rates. SIRs above 1 indicate that the incidence of RRT for a SOA is greater than expected based on the Northern Ireland’s average age-specific incidence rates.

The UKRR reports data for nine different identifiable primary renal diseases (PRD)—diabetes, glomerulonephritis, hypertension, polycystic kidney disease, pyelonephritis, renal vascular disease, other, missing and uncertain aetiology. While it is acknowledged that to some extent the PRD of uncertain aetiology may reflect variation between clinicians and centres, for example, in terms of definitions of renal vascular disease and hypertensive renal disease which remain relatively subjective (UK Renal Registry (UKRR) 2016), this cannot account for the significant percentage of cases of uncertain aetiology recorded. A revised coding system was introduced in 2016 which allowed clinicians to indicate the basis for the diagnosis of the renal disease (e.g. based on histology or not). This has reduced the possibility for over-recording of patients as having uncertain aetiology. While diabetes remains the most common identifiable PRD for patients starting RRT, there remains a significant number of patients on the UKRR identified with uncertain aetiology (e.g. 29.4% of patients’ incident to RRT in 2017 were identified to diabetes and 14.9% to uncertain aetiology, UKRR 2019). CKDu is a global issue, and while the underlying causes have been linked to environmental factors, they are not well understood (WHO 2016). Therefore, the underlying causes of CKDu, as reported in the UKRR, require further investigation. Previous research using UKRR and environmental data is limited (e.g. Jackson et al. 2016; McKinley et al. 2020) and highlights the need for further research in this area. This is the first study, to the authors’ knowledge, which explores the underlying causes of CKDu using social deprivation and environmental factors.

**Fig. 1:**
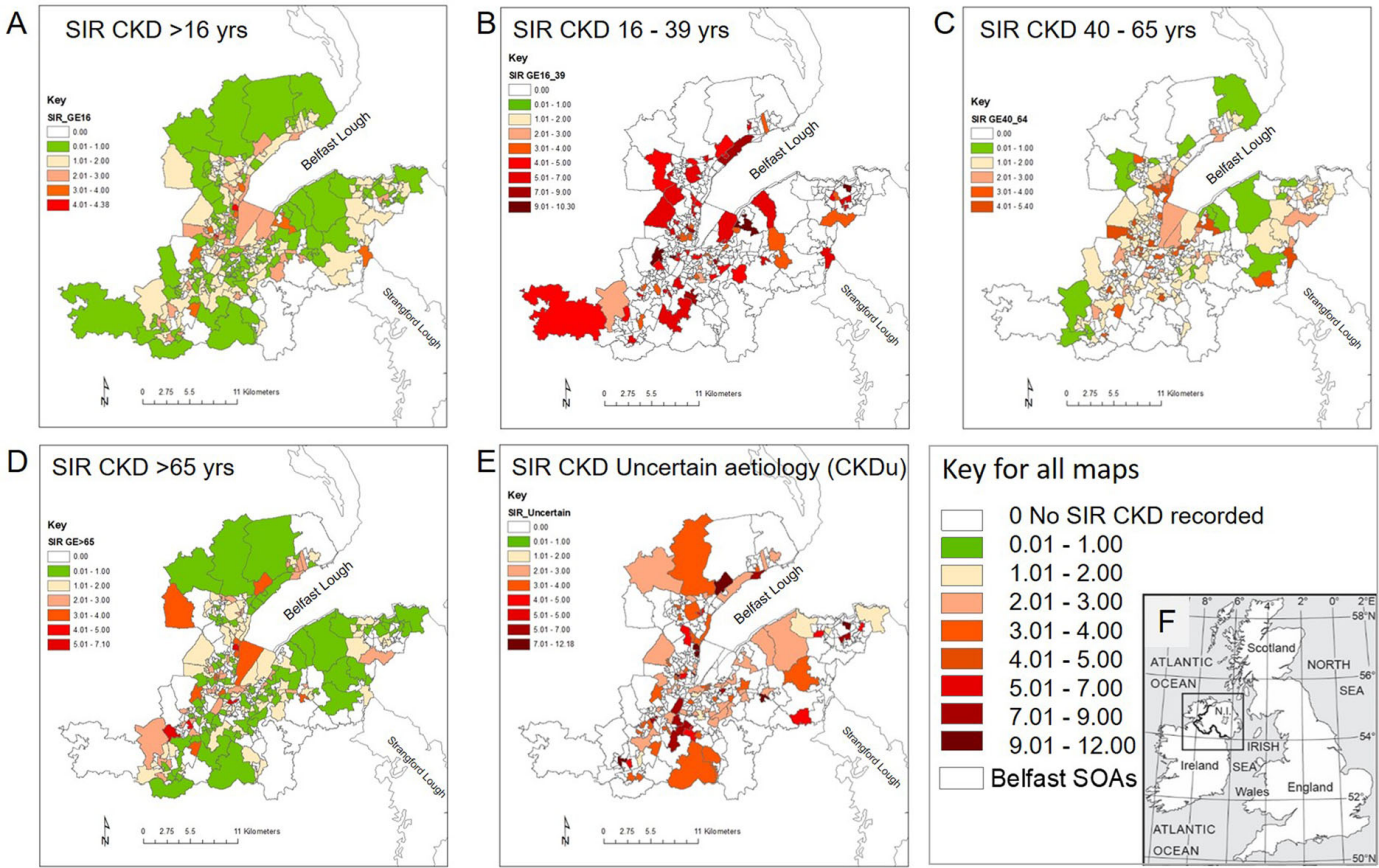
Standardised incidence rates (SIRs) for patients starting renal replacement therapy (RRT) between 2006 and 2016, by Super Output Areas (SOAs) for the greater Belfast area. The SIRs for chronic kidney disease (CKD) were provided by the UK Renal Registry (UKRR) in age brackets: (**a**) all ages > 16 years; (**b**) 16–39 years; (**c**) 40–64 years; (**d**) > 65 years; and (**e**) for chronic kidney disease of uncertain aetiology (CKDu). The key for all maps. (**f**) Location inset map

**Fig. 2:**
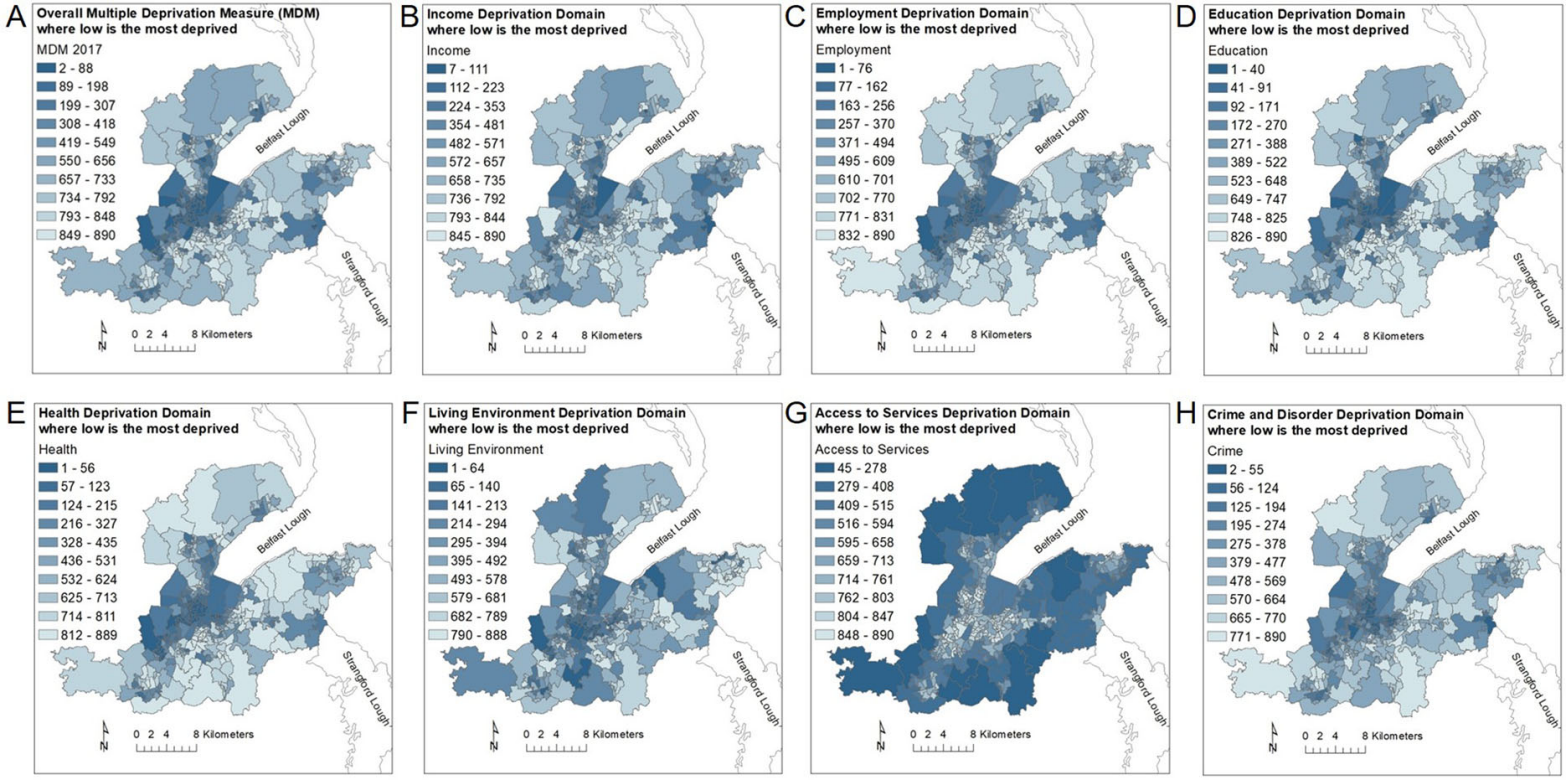
Northern Ireland Multiple Deprivation Measures (MDMs) for 2017 for all Belfast urban Super Output areas (SOAs) including information on (**a**) an overall MDM ranking and (**b**–**h**) seven individual domains of deprivation across the greater Belfast area. MDM data provided by Northern Ireland Statistics and Research Agency (NISRA 2017) Colour scheme conforms with NISRA

For this study, we focused on the greater Belfast urban area. Of the 265 SOAs within the greater Belfast area, 92 SOAs show SIRs for CKDu (Fig. 1, Table 2). Within the urban Belfast area, several SOAs show SIRs for CKDu up to 12 times greater than expected relative to Northern Ireland’s average incidence rate (Fig. 2). This statistic highlights the need for the current study since these elevated incidence rates of CKDu cannot be explained by the known causes of CKD.

**Table 2:** Summary statistics for UKRR standardised incidence rates (SIRs) for patients starting renal replacement therapy (RRT) between 2006 and 2016, by Super Output Areas (SOAs) provided in age brackets, 16–39, 40–64 and 65 + , all ages > 16 and for chronic kidney disease of uncertain aetiology (CKDu) for the greater Belfast area. Data provided by UK Renal Registry (UKRR)

CKD SIR	>16	>16–39	40–65	>65	CKDu
Min	0.27	2.20	0.7	0.3	1.69
1st Qu.	0.60	3.90	1.30	0.80	2.50
Median	1.10	4.60	1.60	1.10	3.30
Mean	1.25	5.03	1.99	1.53	3.75
3rd Qu.	1.60	5.70	2.50	1.80	4.00
Max	4.40	10.3	5.40	7.40	12.18
SOA count	265	66	166	218	93

### Measurement of social deprivation

Social deprivation was measured using multiple deprivation measures provided by the Northern Ireland Statistics and Research Agency (NISRA 2017). The Northern Ireland Multiple Deprivation Measures (MDMs) for 2017 provided information on seven individual domains of deprivation across the greater Belfast area and an overall MDM ranking (Fig. 2). The ranking scale was from 1 which represents the most deprived to 890 for the least deprived. The individual domains, with their relative contribution to the overall MDM, comprised income (25%), employment (25%), health deprivation and disability (15%), education, skills and training (15%), access to services (10%), living environment (5%) and crime and disorder (5%). The individual domains of deprivation and the overall MDM rankings can be used to examine the spatial distribution of the relative deprivation of each SOA and explore any observed association with CKD SIRs. The social deprivation measures of income, employment and education have been used as an indication of socio-economic factors such as smoking (Layte and Whelan 2009), which has been cited to have a detrimental effect on the kidneys (Afsar et al. 2019).

### Environmental factors (geogenic and anthropogenic)

**Fig. 3:**
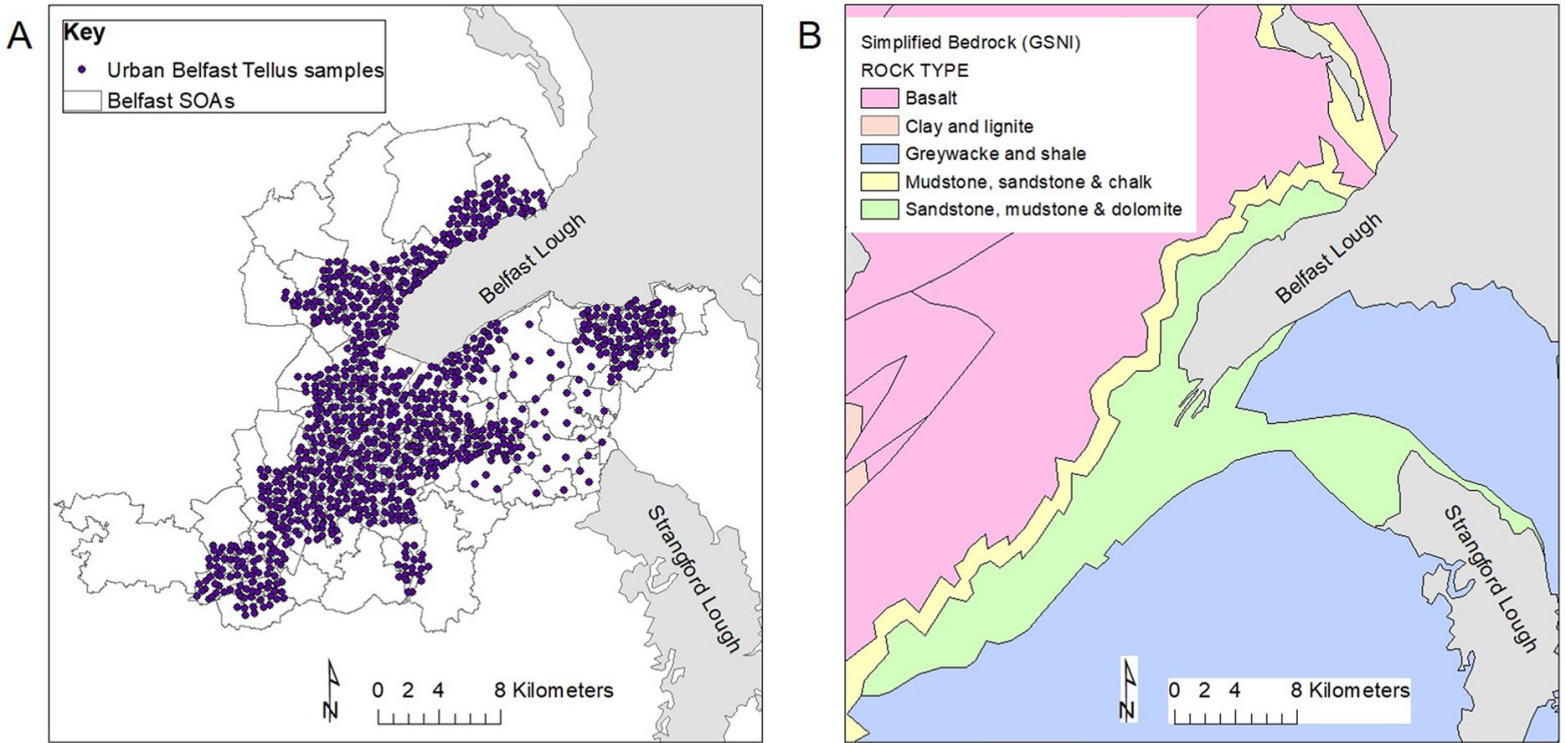
(**a**) Sample locations for the Tellus project (Young and Donald 2013) urban soil geochemical data for the greater Belfast area, Northern Ireland. The Super Output Areas (SOAs) for Belfast—the smallest administrative areas are also shown. (**b**) Simplified geology of the greater Belfast urban area (provided by Geological Survey of Northern Ireland (GSNI), Mitchell 2004)

We used an urban geochemical database generated as part of the Tellus Survey (Young and Donald 2013). The database consists of 1164 soil samples collected across the greater Belfast urban area of Northern Ireland, UK, and analysed for 58 elements with XRF elemental analysis (Fig. 3a). The collection and analysis methods for urban soils used a sampling density of four sites per square kilometre with sampling sites corresponding closely to a predefined grid which did not avoid areas of human influence. Full details on the data collection and analysis techniques are provided in Young and Donald (2013). The Belfast urban area is underlain by several different rock types (Fig. 3b; Mitchell 2004; Young and Donald 2013). The oldest rocks consist of Silurian greywacke and shales which are overlain by Permo-Triassic sandstones and mudstones, and in the west of the urban area by Cretaceous sandstone and chalk. The north-west of the urban area lies on the boundary of the Palaeocene flood basalts dominant over the north west of Northern Ireland. Glacial till and sands form superficial alluvium deposits in the vicinity of the main river, the River Lagan, a legacy of the glacial history of Northern Ireland. Bedrock and superficial geology have been shown to be an important environmental factor as a geogenic source of potentially toxic elements (PTEs) in soils across Northern Ireland (Barsby et al. 2012; Cox et al. 2013; McKinley et al. 2013; McIlwaine et al. 2014, 2015; Palmer et al. 2015). The Silurian shales show elevated As and Mo, while Palaeogene basalts have elevated concentrations for a range of PTEs including chromium (Cr), cobalt (Co), Cu, nickel (Ni), vanadium (V) and Zn.

Anthropogenic contamination in urban areas has been found to have an increasing impact on urban soils resulting in elevated levels of PTEs (McIlwaine et al. 2017). In Belfast, the industrial legacy of ship building, including associated industries such as brass production, along with the more recent development and expansion of Belfast City airport, have been cited as potential anthropogenic sources for urbanisation-based PTEs including Sn, Sb and Pb (linked to ship building) and Zn and Cu attributed to brass production (Herting et al. 2008). As a result, urbanisation-related PTE tracers for Belfast can be split into two main groups explained by geogenic (Co, V, Cr and Ni) and anthropogenic (Zn, Sn, Pb, Sb, As and Mo) factors (McIlwaine et al. 2017; McKinley et al. 2020). Informed by the literature on potential environmental links with CKDu including geogenic and anthropogenic factors, 10 geochemical PTEs (Co, V, Cr, Ni, Zn, Sn, Pb, Sb, As and Mo) were selected for this study. Geochemical data below detection limit were imputed using the detection limits provided in Young and Donald (2013). The soil geochemistry data (1001 sample points) were linked to the 265 SOAs and MDMs within the greater Belfast urban area. This provided 1001 sample points including zero-inflated SIRs of CKD, 899 sample points for SIRs of CKD without zeros and 340 sample points for SIRs of CKDu without zeros.

## Methods

**Fig. 4:**
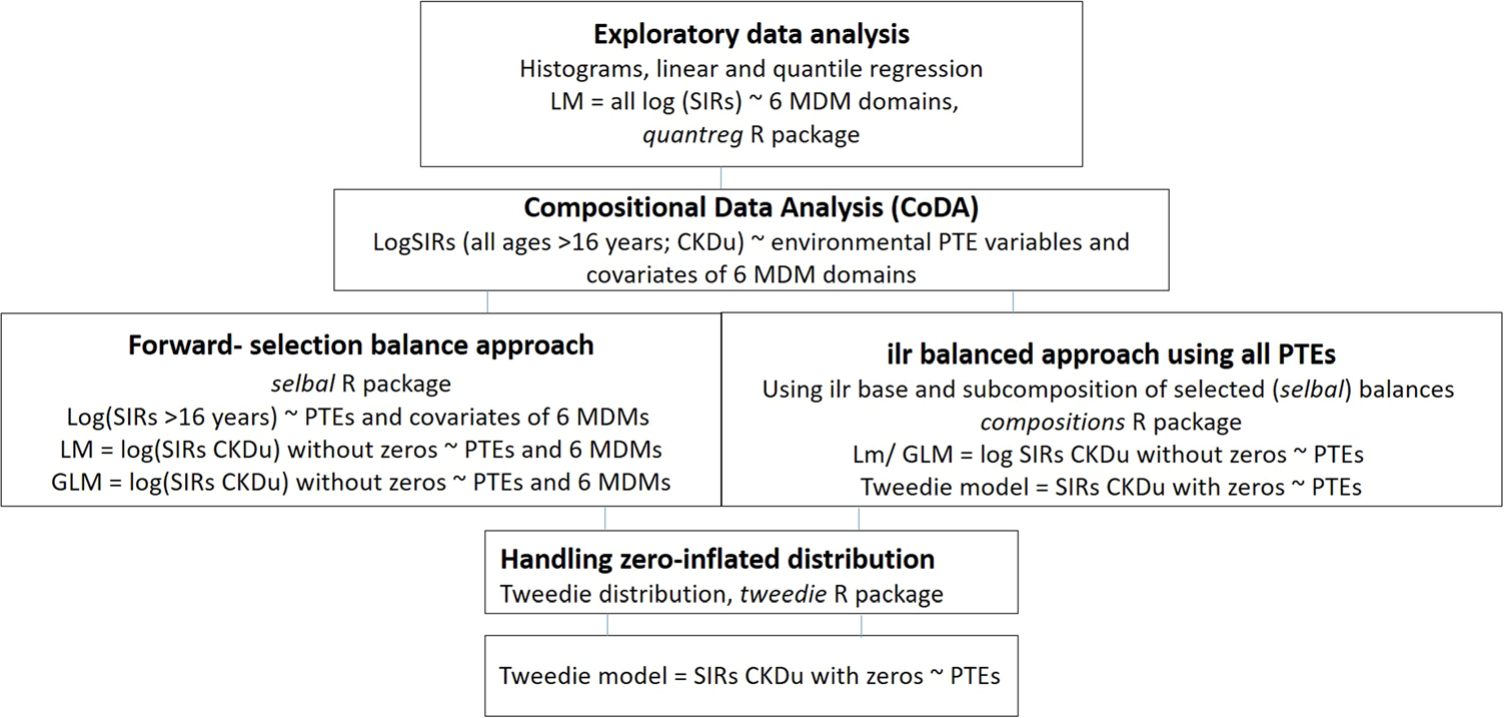
Data analysis approach used in the study. Abbreviations: linear model (LM); generalised linear model (GLM); standardised incidence rates (SIRs) for chronic kidney disease (CKD); chronic kidney disease of uncertain aetiology (CKDu); log-transformed SIRs (log SIRs); multiple deprivation measures (MDMs); potentially toxic elements (PTEs). Note log-transformed SIRs were not used for the Tweedie model, as the aim was to test the impact of the zeros on the relationship with the PTEs

The statistical approach used to explore the relationship between the SIRs of CKD and CKDu with social deprivation measures and with environmental factors, using the urban soil geochemistry database, is shown in Fig. 4 and described in more detail in the sections below. The analysis involves several stages of investigation as outlined below.

1. The MDM rankings are used initially to explore any observed association with CKD SIRs and the relative deprivation of each SOA.
2. Social deprivation measures of income, employment and education are used as an indication of socio-economic factors such as smoking to examine any observed association with CKD SIRs.
3. Quantile regression is applied to quantify the above associations.
4. The influence of anthropogenic and geogenic environmental factors is investigated through the use of urbanisation-related PTE tracers to explore any relationship with SIRs for CKDu. Since environmental factors and deprivation are linked—more deprived areas are generally found closer to industrial zones and transport networks which may be sources of soil contamination—both MDMs and geochemical PTEs are included in the exploration of associations with SIRs for CKDu.
5. Different compositional approaches are used to account for the compositional nature of the geochemical data.
6. The use of different regression models is explored including linear regression and a generalised linear model (GLM).
7. The impact of SOAs with no reported SIR data (zero-inflated data) on the results is investigated through the use of a Tweedie model.

### Exploratory data analysis

Initial exploratory data analysis included the use of linear and quantile regression to examine any potential relationship between CKD SIR for the different age brackets (16–39, 40–64 and 65 + , all ages > 16) and CKDu, and the MDMs. The *quantreg* R package (Portnoy and Koenker 1989; Koenker 2005) was used. Quantile regression provided the opportunity to study the impact of the overall MDM ranking and the seven individual domains of deprivation for Belfast SOAs on different quantiles of the dependent variable’s distribution (in this case the SIRs), to provide a more complete picture of the relationship between CKD including CKDu and social deprivation (socio-economic factors which may be used as a proxy for factors such as smoking). Log-transformed CKD SIRs were used for this stage of the analysis.

### Compositional data analysis and use of balances

A balance in compositional data analysis (CoDA) corresponds to the difference in means of the log-transformed abundances between two subcompositions (Egozcue et al. 2003, Egozcue and Pawlowsky-Glahn 2005, 2011). Two approaches were explored to identify components (in this study MDMs and geochemical PTEs) whose relative abundance is associated with elevated incidences of CKD (Fig. 4). The first was a forward selection method using the *selbal* algorithm first proposed by Rivera-Pinto et al. (2018). The approach using geochemical data to identify an elemental balance with the best association with CKDu (unknown aetiology) is described in more detail in McKinley et al. (2020). The *selbal* selection procedure searches for a signature in terms of a balance between two groups of parts that adequately explains the response variable of interest. A fivefold cross-validation (CV) procedure was used to identify the set of balances. An associated regression model was used to calculate the mean response based on the balances identified in the parameter estimation step. The mean squared error (MSE) as a function of the number of components included in the balance indicates the optimal number of variables identified in the forward selection process whose relative abundance is associated with the response variable of interest. The second approach was a compositional isometric log-ratio (ilr) approach based on a sequential binary partition (SBP) using the R package *compositions* (Van den Boogaart and Tolosana-Delgado 2008).

Using the two compositional balance approaches: log-transformed (using selbal cross-validation option) and ilr-transformed (using an SBP approach) environmental PTE variables (Co, V, Cr, Ni, Zn, Sn, Pb, Sb, As and Mo) and covariates of six individual domains of deprivation MDMs (comprising income, employment, health deprivation and disability, education, skills and training, access to services, and living environment) were used to identify the elemental balance with the strongest association with log-transformed SIRs of CKD for all ages > 16 years and CKDu (unknown aetiology) using the 2006–2016 UKRR SIR data for the Belfast urban area.

### Zero-inflated distribution

**Fig. 5:**
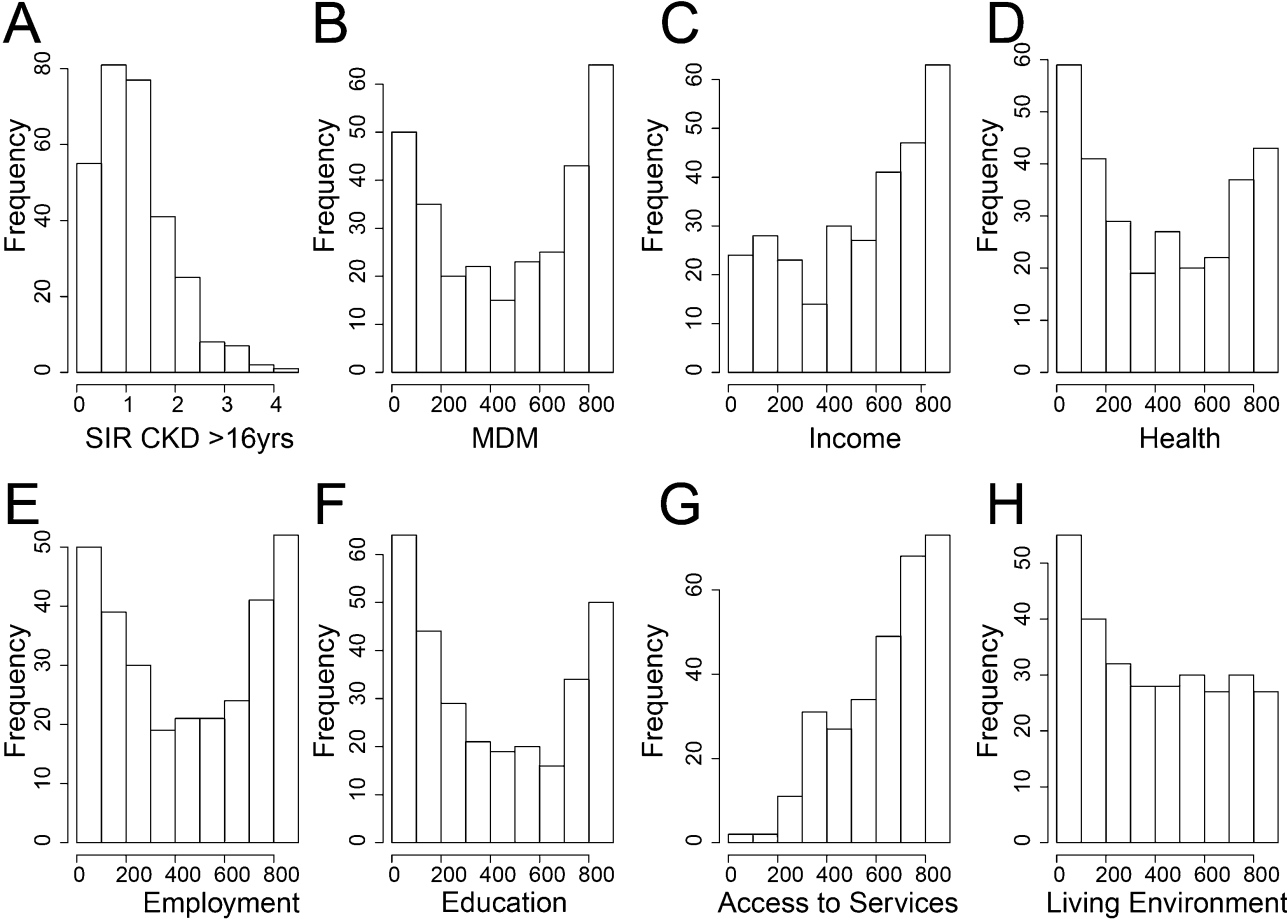
Histograms showing distribution of Super Output Areas (SOAs) with (**a**) standardised incidence rates (SIRs) of chronic kidney disease (CKD) for all ages > 16 years along with (**b**) overall multiple deprivation measure (MDM) ranking and six individual domains of deprivation comprising (**c**) income, (**d**) health deprivation and disability, (**e**) employment, (**f**) education, skills and training, (**g**) access to services and (**h**) living environment. The ranking scale is from 1 which represents the most deprived to 890 for the least deprived

SIRs are measures that indicate the relationship between expected incidence and actual incidence. The UKRR data include multiple SOAs with zeros corresponding to no reported incidences of CKD for the different age groups of CKDu. Zeros may also correspond to SOAs where the number of incidences of CKD is too small (and, therefore, has been suppressed to avoid the risk of re-identification of patients; the UKRR guidelines indicate that any cells with *n *≤ 5 should be suppressed) (UKRR Information sharing Protocol, UKRR 2019). In both cases, SOAs with zeros may be meaningful to provide an increased understanding of the environmental factors associated with CKDu. Therefore, given the zero-inflated nature of the data, the skew of the distribution of the SIRs for CKD (Fig. 5a) and non-negativity of the data, other regression models were investigated and a Tweedie model (Tweedie 1984) was deemed to be appropriate to examine the influence of including SOAs with no incidences of CKD for all ages above 16 years and CKDu. The Tweedie distribution is a special case of an exponential distribution which allows for a cluster of data values at zero and a mixture of zeros and non-negative data points (Jorgensen 1987, 1997). The R package *tweedie* was used which produces a generalized linear model family object with a power variance function and a power link (Dunn and Smyth 2005, 2008). Note that SIRs were not log-transformed for the Tweedie model, as we wanted to test the impact of the zeros on the relationship with the PTEs.

## Results

**Fig. 6:**
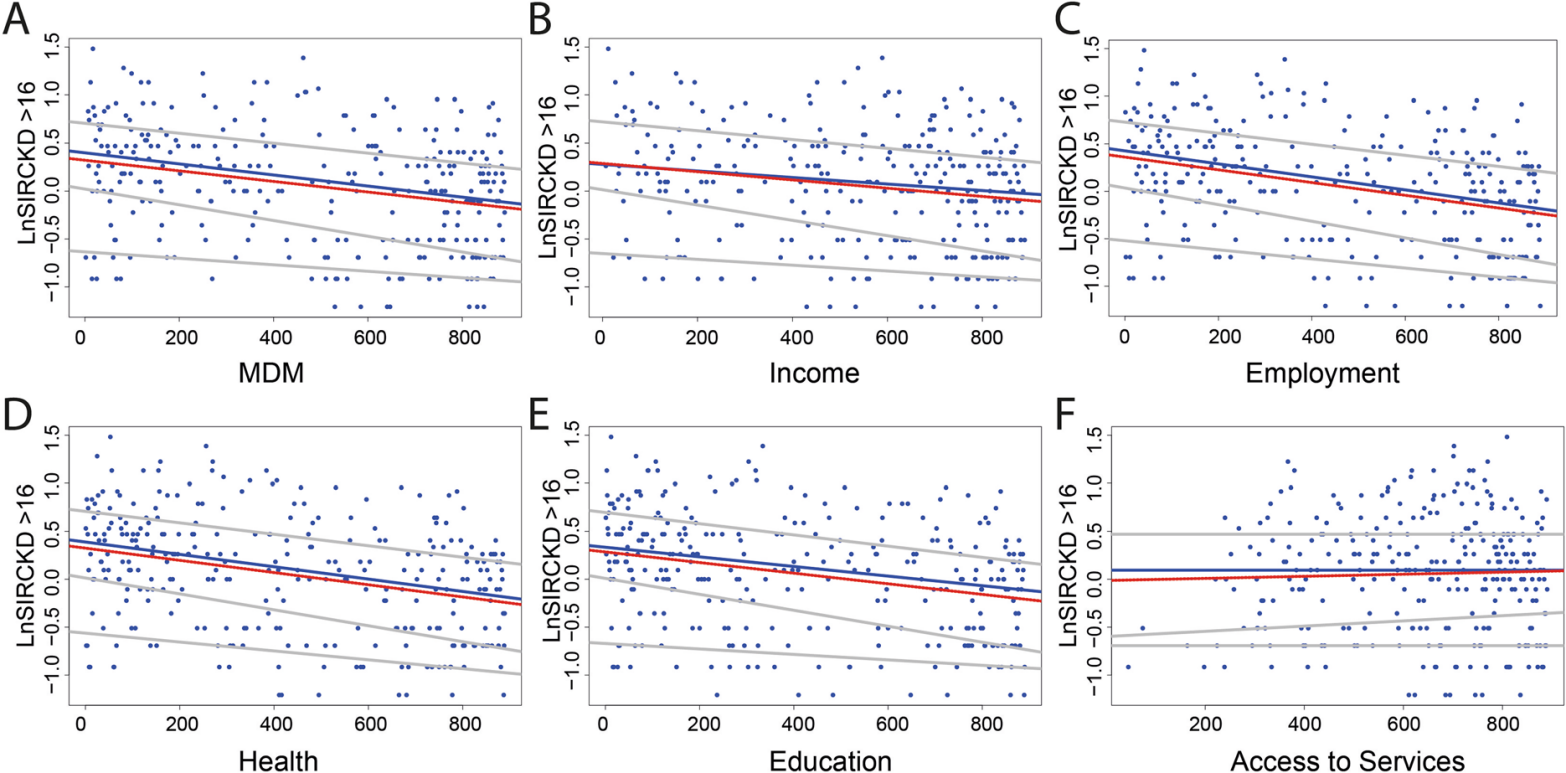
Scatter plots of standardised incidence rates (SIRs) of chronic kidney disease (CKD) for all ages > 16 years with overall (**a**) multiple deprivation measures (MDMs) ranking and the individual domains of deprivation (**b**) income, (**c**) employment, (**d**) health, (**e**) education and (**f**) services. Lines show linear regression (red line) and quantile regression (blue lines) for 0.25, 0.5 and 0.75 quantiles

Exploratory data analysis was used to reveal any relationship between CKD and social deprivation using the multiple deprivation measures (MDMs). The spatial maps (Fig. 2) and histograms (Fig. 5b–h) showing the frequency distribution of the overall MDM and six individual domains of deprivation MDMs (comprising income, employment, health deprivation and disability, education, skills and training, access to services, and living environment) are shown for SOAs across the greater Belfast urban area. Several deprivation MDM domains such as health, employment and education show similar spatial patterns and a bimodal distribution with a large number of SOAs with lower rankings (< 10) and a high frequency of high MDM rankings (> 800). This reflects the occurrence in Belfast of both the most deprived SOAs and the least deprived SOAs in Northern Ireland. In this exploratory stage, linear regression was conducted to investigate the relationship between the log-transformed SIRs and the MDMs. A statistically significant relationship is shown between SIR CKD for all ages > 16 yrs and income and employment (to a significance level of 0.01 and 0.001, respectively), between SIR CKD 40–64 yrs and income (to a significance level of 0.01) and between SIR CKD > 65yrs and income, employment and health (to a significance level of 0.01, 0.01 and 0.001, respectively; Table 1 supplementary material). However, it is clear that the relationship is complex and linear regression is insufficient to describe fully the relationship between the SIRs and MDMs. To elucidate the relationship further, quantile regression was used to investigate the impact of the relationship between the SIR distributions and different quantiles of the overall MDM ranking and six individual domains of deprivation for Belfast. Although quantile regression between the logged SIRs and MDMs indicates the lack of a large correlation (Fig. 6), the general trend indicates that there is a small negative correlation between CKD and the overall MDM and the domains of income, employment, health (deprivation and disability), education and skills and training. A small positive correlation is indicated between CKD and access to services suggesting an increased opportunity for diagnosis with greater access to services. Overall, the results suggest that there may be a negative relationship between the SIRs of CKD and the social deprivation measures of income, employment, health and education. The most deprived SOAs (lowest MDM rankings) are associated with the largest SIRs of CKD, whereas the least deprived SOAs show smaller SIRs of CKD. The MDMs of income, employment and education were used as proxies of socio-economic factors such as smoking. In this respect, it could be inferred that the results indicate an association between larger SIRs of CKD and the socio-economic factors used as a proxy for lifestyle behaviours such as smoking. However, the results indicate that the MDMs are insufficient to explain fully the distribution of SIRs of CKD.

The next stage of the analysis investigated the influence of anthropogenic and geogenic environmental factors through the use of urbanisation-related PTE tracers to explore if a relationship is found between the SIR for CKDu and PTEs. It was deemed important and relevant to include the MDMs in the analysis to investigate any potential associations with the SIR for CKD and CKDu. The rationale is that it can be argued that anthropogenic environmental factors and deprivation may interact in that more deprived SOAs are generally found closer to past and current industrial zones and transport networks including road, rail and, in this case, Belfast City airport. These anthropogenic factors have been shown to act as sources of soil contamination and are reflected in the urbanisation PTE tracers. Compositional data analysis was used through different balance selection approaches: (1) a forward selection method using the *selbal* algorithm; (2) an ilr approach using all PTEs; and (3) an ilr subcomposition approach informed by the selected balances from *selbal*. Once the balances were identified through these approaches, the strength of relationship was explored through linear regression (LM) and generalised linear modelling (GLM with log-link). Initially, the analyses were conducted excluding SOAs with no incidences of CKD and CKDu (zeros). Following this, the effect of zero inflation was explored through the Tweedie model including SOAs with no SIRs for CKD or CKDu. A summary of all results is provided in Table 3 (with details of coefficients and graphs—residual vs fitted; normal Q–Q plot; scale location and residuals vs leverage—provided in supplementary material Figs. 1–4).

**Table 3:** Summary of regression results for standardised incidence rates (SIRs) for chronic kidney disease (CKD) for all ages > 16 years and for SIRs CKD of uncertain aetiology (CKDu), multiple deprivation measures (MDMs) income, employment, education, health, living and services. Please see Supplementary Material Tables 2–5 and Figs. 1–4 for full details of coefficients and graphs. See Supplementary Material Table 5 for full details of ilr base balances

Balance Selection	Dependent variable	Model	Balance	MDMs	Statistical significance
Selbal global balance only	lnSIR > 16	GLM	Sb/Sn	All	Sb/Sn (0.05), Employment (0.001), Income (0.01), Education (0.01), Services (0.01)
	lnCKDu (without zeros)	LM	Cr/Ni	All	Cr/Ni (0.001), Living (0.001)
		GLM	Cr/Ni	All	Cr/Ni (0.001), Living (0.001)
	CKDu (with zeros)	Tweedie (link powe*r* = 0)	Cr/Ni	All	Cr/Ni (0.05)
Selbal all balances	lnSIR > 16	GLM	Sb/Sn, Co/Ni, Pb/Sb	All	Sb/Sn (0.05), Co/Ni (0.1), Employment (0.001), Income (0.01), Education (0.01), Services (0.01)
	lnCKDu (without)	LM	Cr/Ni, As/Mo, Co/Ni	All	Cr/Ni (0.001), Living (0.001)
		GLM	Cr/Ni, As/Mo, Co/Ni	All	Cr/Ni (0.001), Living (0.001)
	CKDu (with zeros)	Tweedie (link powe*r* = 0)	Cr/Ni, As/Mo, Co/Ni	All	Cr/Ni (0.05), As/Mo (0.05), Co/Ni (0.1)
ilr base approach	CKDu (with zeros)	Tweedie (link powe*r* = 1)	ilr base (see Supplementary Table 5)		ilr 4 (Mo/As,Cr,Co; 0.01), ilr 5 (Ni/As,Co,Cr,Mo; 0.05) Also large balances ilr 8 and ilr 10
ilr-balanced approach using subcomposition	CKDu (with zeros)	Tweedie (link powe*r* = 0)	Cr/Ni, As/Mo, Co/Ni		

**Fig. 7:**
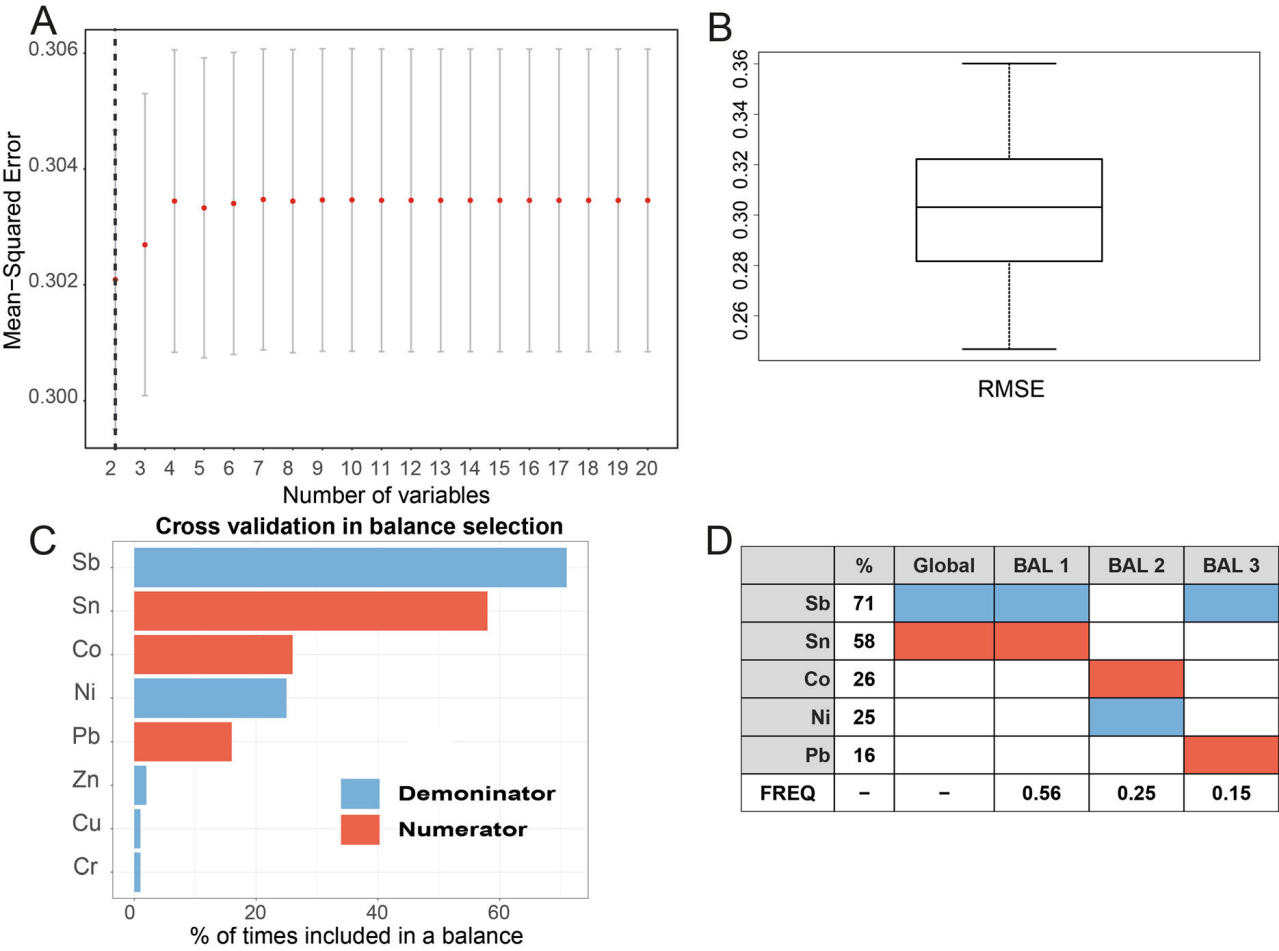
Results of the forward selection method using the *selbal* algorithm (899 sample points) shown for Belfast urban area (Super Output Areas (SOAs)) standardised incidence rates (SIRs) of chronic kidney disease (CKD) for all ages > 16 years with soil PTEs and six individual domains of deprivation multiple deprivation measures (MDMs—comprising income, employment, health deprivation and disability, education, skills and training, access to services, and living environment), (**a**) mean squared error (MSE) as a function of the number of components included in the balance. The optimal number of components is highlighted with a vertical dashed line; (**b**) box plot of root-mean-squared error (RMSE); (**c**) the balance identified with the whole data set is the most frequently identified in the cross-validation (CV) procedure; and (**d**) global balance and other balances identified in CV procedure

**Fig. 8:**
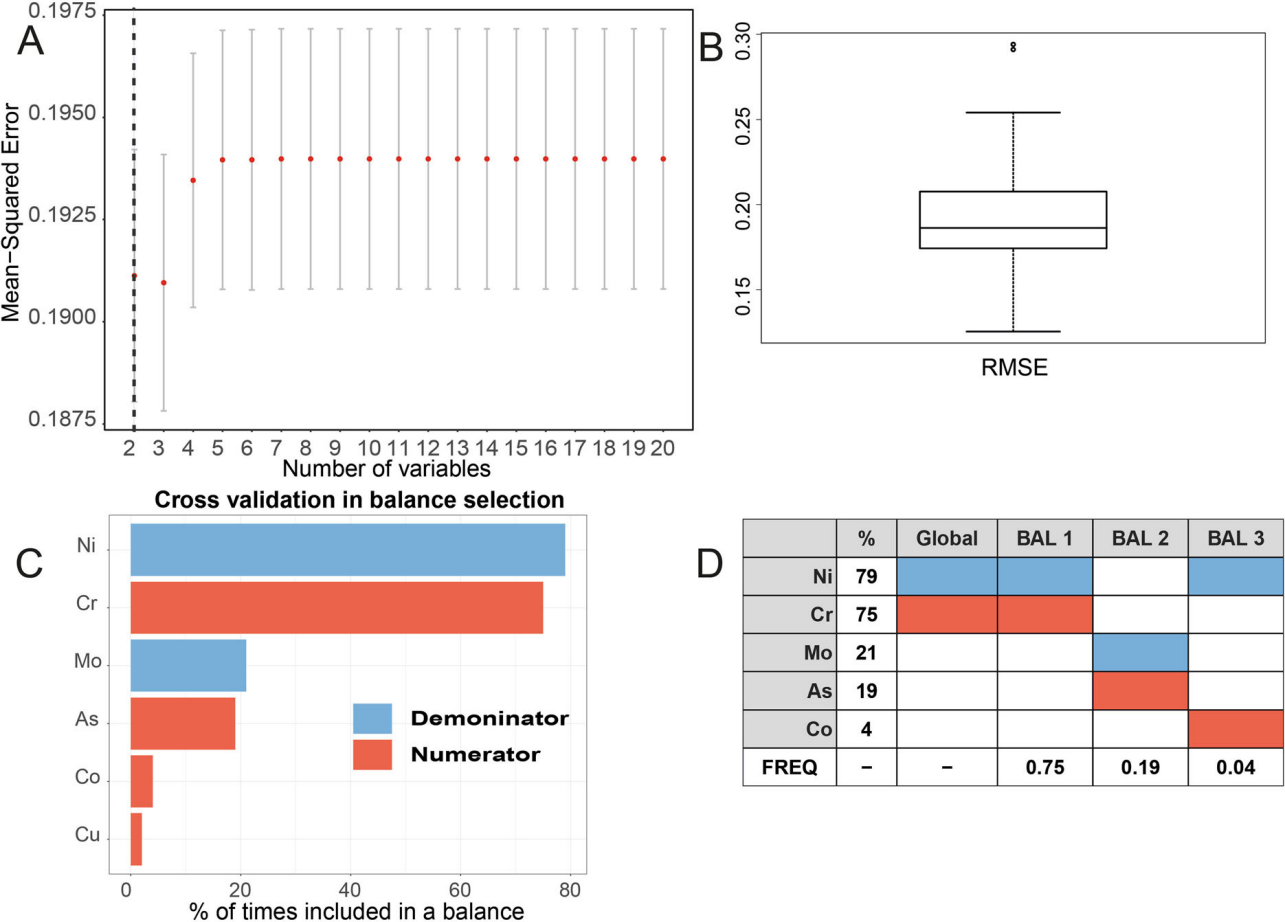
Results of the forward selection method using the selbal algorithm shown for Belfast urban area (340 sample points) standardised incidence rates (SIRs) of chronic kidney disease of uncertain aetiology (CKDu) with soil PTEs and six individual domains of deprivation multiple deprivation measures (MDMs comprising income, employment, health deprivation and disability, education, skills and training, access to services, and living environment), (**a**) mean squared error (MSE) as a function of the number of components included in the balance. The optimal number of components is highlighted with a vertical dashed line; (**b**) box plot of root-mean-squared error (RMSE); (**c**) the balance identified with the whole data set is the most frequently identified in the cross-validation (CV) procedure; and (**d**) global balance and other balances identified in CV procedure

The results of the forward selection method using the *selbal* algorithm are shown for the log-transformed SIRs of CKD for all ages > 16 years (Fig. 7 including 899 sample points) and for log-transformed SIRs CKDu (Fig. 8 including 340 sample points). The covariates include the soil PTEs (Co, V, Cr, Ni, Zn, Sn, Pb, Sb, As and Mo) and six individual domains of deprivation MDMs (income, employment, health deprivation and disability, education, skills and training, access to services, and living environment). The elements most frequently identified in the cross-validation procedure, as being most associated with log(CKD) for all SIRs > 16 years, are Sb appearing 71% and Sn appearing 58% of the time, respectively (Fig. 7c, d). They form the balance termed the global balance in *selbal.* In addition to the global balance of Sn/Sb, the balances of Co/Ni and Pb/Sb are also identified in the cross-validation procedure. The regression results suggest a negative relationship of log (CKD SIR > 16) with the MDM domains of employment and income, a slight positive relationship with services (significance levels of 0.001, 0.01 and 0.01, respectively) and a positive correlation with the identified global balance of Sn/Sb (Fig. 6, Table 3; Supplementary material Table 2; significance level 0.05). The results for log(CKDu) identify the balances Cr/Ni(global balance), As/Mo and Co/Ni most frequently in the cross-validation procedure (Fig. 8). Regression results (using LM and GLM) suggest a correlation between log(SIR of CKDu) and the MDM domain of living (significance level of 0.001) and a correlation with the identified balances of Cr/Ni and As/Mo (Table 3; Supplementary material Table 3; significance levels 0.001 and 0.1, respectively).

Exploring the use of a Tweedie model and the effect of zeros on the analysis (SOAs with no SIR for CKDu) confirms a correlation between CKDu and the balances of Cr/Ni, As/Mo and Co/Ni (Summary Table 3 and supplementary material Table 4; significance levels 0.05, 0.05 and 0.1, respectively). Using the *compositions* ilr base approach with all of the PTEs, a significant correlation was observed between CKDu and the balances of Mo/(As,Cr,Co) and Ni/(As,Co,Cr,Mo) (Table 3; supplementary material Table 5; significance levels of 0.01 and 0.05, respectively). Using the balances selected through the *selbal* approach to inform a subcomposition for an ilr-balanced approach, the findings remain consistent, with a statistical correlation between CKDu and the balance of As/Mo (Summary Table 3 and Table 5 supplementary material; significance level 0.05).

## Discussion

The SIRs for CKD for the urban area of Belfast were up to four times greater than expected relative to Northern Ireland’s average incidence rate for CKD (Fig. 2). The high rates of CKD recorded for Northern Ireland correspond with the UK wide and global increasing trend in CKD, and, as such, CKD is likely to place increasing pressure on local health services. Although diabetes and hypertension are recognised as the predominant risk factors for CKD, this study indicates that some of the largest SIRs of CKD are found within the most deprived SOAs in Belfast and a significant negative relationship was observed with the social deprivation measures of employment, income and health (significance levels of 0.001, 0.01 and 0.001, respectively). As these indicators are used as a proxy for smoking (Layte and Whelan 2009), these findings appear to support the cited association between CKD and smoking (Afsar et al. 2019). Belfast also shows SIRs of CKDu up to 12 times greater than expected relative to Northern Ireland’s average incidence rate for CKDu. Although it is reasonable to assume that the PRD of uncertain aetiology may to some degree reflect variation in diagnosis, the findings from this research indicate that the influence of environmental factors that may cause CKDu should not be discounted. Known environmental nephrotoxins include Pb, Cd, Hg and As. Urbanisation through industrialisation, atmospheric pollution deposition, traffic pollution and brake wear emissions have also been linked to harmful impacts on kidney function. Understanding the role of toxic metals through these sources is crucial to understanding the impact of environmental factors, including anthropogenic and geogenic PTEs, on the occurrence of CKDu. This study used a compositional balance approach which complies with the compositional nature of the geochemical soil data and enabled the association of PTEs with CKDu to be explored. Environmental factors and deprivation are intrinsically linked in that more deprived SOAs may be expected to be found closer to industrial zones and transport networks, which may be sources and pathways of soil contamination. Therefore, both MDMs and geochemical PTEs were included in the exploration of associations with SIRs for CKDu.

**Fig. 9:**
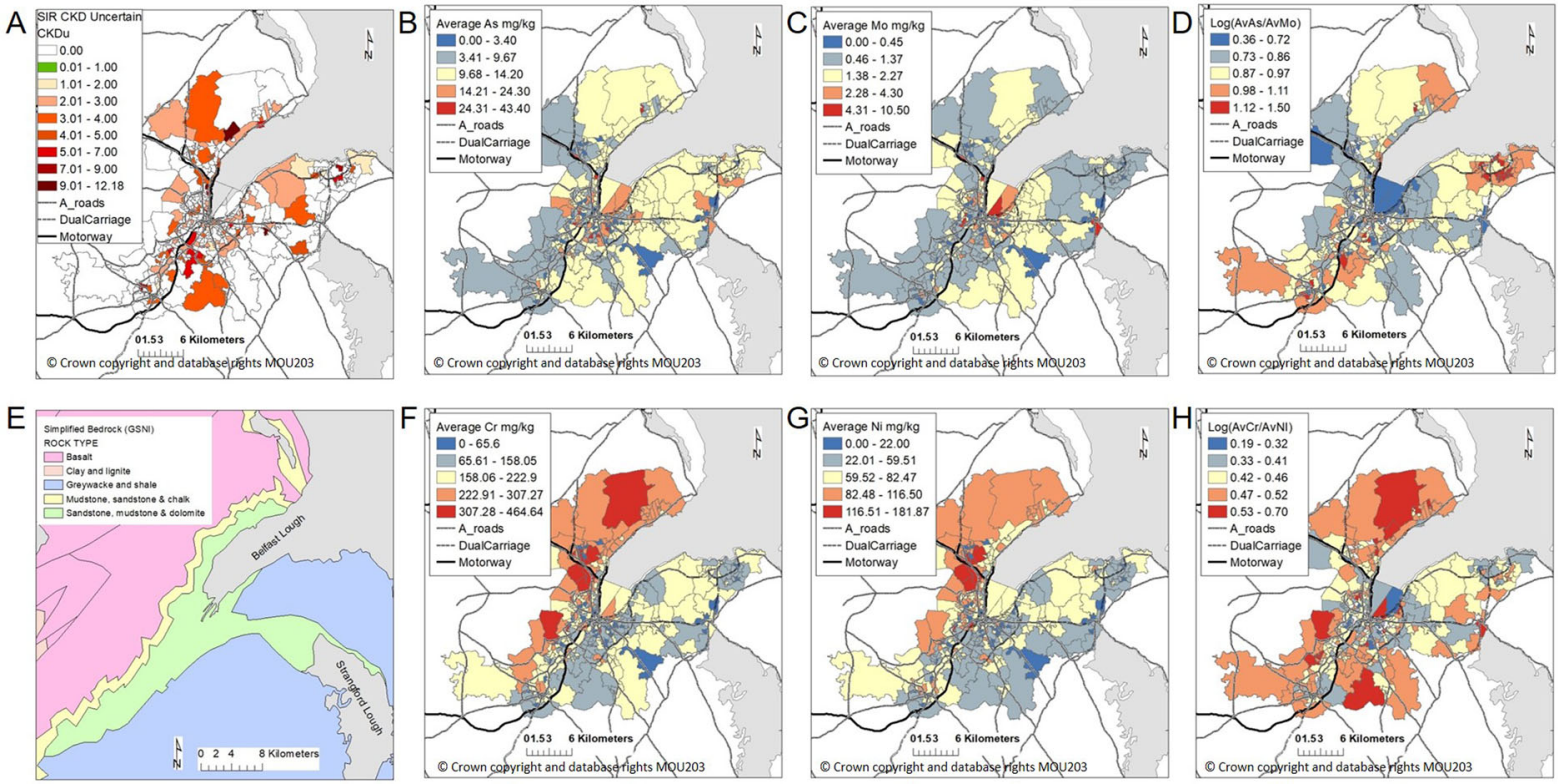
Visualisation of average potentially toxic elements (PTEs) data for SOAs shown with the road network for interpretation purposes. Transport networks reproduced from Land and Property Services data with the permission of the Controller of Her Majesty’s Stationery Office, © Crown copyright and database rights MOU203. (**a**) Standardised incidence rates (SIRs) of chronic kidney disease of uncertain aetiology (CKDu); (**b**) average As mg/kg; (**c**) average Mo mg/kg; (**d**) balance log (As/Mo); (**e**) simplified geology provided by Geological Survey of Northern Ireland (GSNI), Mitchell 2004; (**f**) average Cr mg/kg; (**g**) average Ni mg/kg; (**h**) balance log(Cr/Ni)

The findings highlight several balances of interest. A correlation between CKD SIRs > 16 years and the balance Sn/Sb was identified (significance level 0.05). The balances of PTEs which showed the largest correlation with CKDu using both compositional approaches (*selbal* forward selection and confirmed through ilr-balanced approach) using all regression methods (LM, GLM and Tweedie model) were Cr/Ni and As/Mo (significance levels 0.001 and 0.1, respectively). A further notable correlation between CKDu and the balance of Co/Ni (summary Table 3; significance level 0.05) was confirmed using the ilr-balanced approach and the Tweedie model with zero-inflated data. A summary of the key findings is provided (Table 4) with comparison maps (Fig. 9) showing the road network and the spatial distribution of SIRs of CKDu, simplified geology and the key PTEs and balances highlighted in the findings, to assist in visualisation and interpretation of the results.

**Table 4:** Summary of findings in relation to source, pathways and receptor model: ^1^McIlwaine et al. (2017); ^2^Carrero et al. (2013); ^3^Grigoratos and Martini (2015); ^4^Barsby et al. (2012); ^5^Palmer et al. (2015); ^6^Geiser (1999); ^7^Oberdörster et al. (2005). Standardised incidence rates (SIRs), chronic kidney disease (CKD), chronic kidney disease of uncertain aetiology (CKDu)

	Source: soil geochemistry compositional balance	Pathway: soil as a tracer for urbanisation pollutants	Receptor: significance for CKD
CKD SIRs > 16 years	Sb/Sn, Co/Ni, Pb/Sb	Sn, Sb and Pb: linked to ship building^1^ Sn, Sb, Pb: Atmospheric pollution deposition, traffic pollution^2^; Brake lining and brake wear emissions^3^ Co/Ni: Elevated levels in Palaeogene basalts^4,5^	Ultrafine particles can become blood-borne and translocate to other tissues such as the liver, brain and kidney^6,7^
CKDu	As/Mo, Cr/Ni, Co/Ni	As, Mo: Atmospheric pollution deposition, traffic pollution^2^; Brake lining and brake wear emissions^3^ As, Mo: Elevated levels in Silurian shales^4,5^	
		Ni, Cr, Ni: Elevated levels in Palaeogene basalts^4,5^

Previous studies showed how weathering of certain types of bedrock has resulted in geogenic PTE contamination of soils across Northern Ireland (Barsby et al. 2012; Cox et al. 2013; McKinley et al. 2013; Jackson et al. 2016; McIlwaine et al. 2014, 2015; Palmer et al. 2015). The spatial distribution of Ni and Cr (Fig. 9) appears to closely correspond to the Palaeogene basalts which are known to have elevated concentrations of these PTEs. The underlying bedrock of Belfast includes Silurian shales (Fig. 9e) known to contain elevated levels of As and Mo. However, these PTEs have also been attributed to anthropogenic sources such as atmospheric pollution deposition including traffic pollution (Carrero et al. 2013) and Mo has been linked to brake lining and brake wear emissions (Grigoratos and Martini 2015). Mapping the road network reveals a variable relationship with SIRs of CKDu (Fig. 9a) and also with elevated incidences of As and Mo. The balance of As/Mo identified in the analysis may illuminate how the sources of Mo and As vary across Belfast in that both As and Mo have been linked to atmospheric pollution deposition including traffic pollution (Carrero et al. 2013), but brake lining and brake wear emissions may be a potential additional increasing source of Mo (review by Grigoratos and Martini 2015). The implications from this study are that PTEs in urban soils may be used as a proxy for the availability of nephrotoxins from environmental pollution and should be considered when investigating explanatory factors for CKDu. The findings from this study which showed a significant correlation between CKDu and these PTEs points to the importance of environmental PTEs linked to urbanisation in understanding the multifactorial explanatory factors for CKDu.

In summary, the results from this research point towards two key findings: First elemental toxins in soil, which may occur as a result of weathering of natural geological bedrock and as a legacy of anthropogenic contamination, pose a risk to human health and show a statistically significant relationship with elevated incidence of CKDu in areas of Belfast. The relationship with CKDu needs to be explored for other urban centres with underlying geology with naturally elevated levels of elemental toxins and/or areas with a historical legacy of anthropogenic contamination. Second soils can act as indicators of atmospheric and traffic pollution, and this research suggests a link between CKDu and nephrotoxins from traffic pollution and brake emissions. Thus, soils can be used as proxies for the contribution from the “environment” to the burden of disease. Further research is needed to refine the relationship between evidence of anthropogenic toxins in soils and air quality information. This would enable greater refinement of the models presented in this research. Moreover, greater knowledge of the temporal relationships between population and the local environment (such as residence time and traffic flow) would provide greater insight into this complex relationship. This research has shed light on the increasing burden of CKD and, in particular, the environmental and anthropogenic factors that may be linked to CKDu, adding to the heterogeneity of causes of progressive renal failure such as diabetes and hypertension. There is great scope to extend this research further both in Belfast and in other cities of the world, particularly where industry has historically been prevalent.

## Conclusions

This research contributes to a greater understanding of the multifactorial causes of CKDu and the heterogeneity of progressive CKD through the use of urban soil geochemistry as indicators of environmental toxins including atmospheric pollution and traffic pollution. A relationship was observed between CKD with the social deprivation measures of employment, income and education. This statistical relationship was most significant for CKD SIRs for all ages above 16 years, SIRs 40-64 yrs and SIR CKD > 65yrs (significance levels of 0.001, 0.01 and 0.001, respectively). Since deprivation is an indicator of socio-economic behaviours including smoking, this result concurs with the cited association between CKD and smoking. Using a compositional balance approach and regression models, including the linear, generalised linear and Tweedie models, it was possible to identify significant associations between CKD SIR > 16 years, CKDu and elemental balances. The balances of PTEs Sn/Sb, Co/Ni and Pb/Sb were found to be most associated with CKD SIRs > 16 years. The balances of PTEs Cr/Ni and As/Mo were most associated with CKDu using all regression models. The sources of these PTEs can be attributed to the naturally occurring elemental toxins in soils (Ni, Cr and Co) and anthropogenic contamination (Sn, Sb, Pb, As, Mo). Atmospheric pollution deposition, traffic and brake wear emissions have been cited as sources for these PTEs, with a blood-borne pathway of ultrafine particles of these PTEs which may translocate to the kidney. Therefore, the findings from this research, which reveal a correlation between CKDu and these PTEs, suggest the need for greater understanding of the link between CKDu and environmental PTEs linked to urbanisation.

## Electronic supplementary material

Below is the link to the electronic supplementary material.

Supplementary material 1 (DOCX 860 kb)
