# Supplementary material for: Chronic kidney disease of unknown origin is associated with environmental urbanisation in Belfast, UK
Source: Environ Geochem Health. 2020 Jun 24;43(7):2597–614. doi: 10.1007/s10653-020-00618-y (PMC8275563; doi:10.1007/s10653-020-00618-y)
Supplement: Supplementary file 1 — Supplementary material 1 (DOCX 860 kb) [file 10653_2020_618_MOESM1_ESM.docx]

Supplementary Material for McKinley et al. EAGH

| Dependent variable |  | Estimate | Std. Error | t value | Pr(>\|t\|) | R-Squared |
| --- | --- | --- | --- | --- | --- | --- |
|  |  |  |  |  |  |  |
| lnSIR CKD>16 yrs | Intercept | 0.2602508 | 0.072913 | 3.569 | 0.000418 | 0.1292 |
|  | Income | 0.0004743 | 0.000203 | 2.34 | 0.019943 |  |
|  | Employment | -0.0010141 | 0.000182 | -5.56 | 6.07E-08 |  |
| lnSIR CKD 40-64 yrs | Intercept | 0.8792545 | 0.06717 | 13.09 | <2e-16 | 0.1361 |
|  | Income | -0.0004244 | 0.000191 | -2.228 | 0.0271 |  |
|  | Employment | -0.0002102 | 0.000176 | -1.198 | 0.2324 |  |
| lnSIR CKD >65 yrs | Intercept | 0.49793 | 0.078645 | 6.331 | 1.18E-09 | 0.09653 |
|  | Income | -0.0003829 | 0.000212 | -1.805 | 0.0723 |  |
|  | Employment | 0.0014957 | 0.00059 | 2.534 | 0.01193 |  |
|  | Health | -0.0017008 | 0.000555 | -3.063 | 0.00244 |  |

Table 1: Summary of regression results showing significant results. Standardised Incidence Rates (SIRs) for Chronic Kidney Disease (CKD) for all ages >16 yrs, 40-64 yrs and >65yrs with Multiple Deprivation domains of income, employment and health.

| Dependent variable | Model |  | Estimate | Std. Error | t value | Pr(>\|t\|) | R-Squared |
| --- | --- | --- | --- | --- | --- | --- | --- |
| lnSIR CKD>16 yrs | GLM | (Intercept) | 0.5903 | 0.09701 | 6.084 | 1.74E-09 |  |
|  |  | Income | 0.000356 | 0.000134 | 2.657 | 0.00803 |  |
|  |  | Employment | -0.00195 | 0.000339 | -5.741 | 1.29E-08 |  |
|  |  | Health | 0.000562 | 0.000366 | 1.535 | 0.12515 |  |
|  |  | Education | 4.92E-05 | 0.000193 | 0.256 | 0.79827 |  |
|  |  | Services | -0.0003 | 0.000108 | -2.778 | 0.00558 |  |
|  |  | Living | 6.86E-05 | 7.67E-05 | 0.895 | 0.37102 |  |
|  |  | Global Balance (Sn/Sb) | 0.09126 | 0.04155 | 2.196 | 0.02834 |  |
|  |  |  |  |  |  |  |  |
|  | LM | (Intercept) | 0.5903 | 0.09701 | 6.084 | 1.74E-09 | 0.2453 |
|  |  | Global Balance (Sn/Sb) | 0.09126 | 0.04155 | 2.196 | 0.02834 |  |
|  |  | Income | 0.000356 | 0.000134 | 2.657 | 0.00803 |  |
|  |  | Employment | -0.00195 | 0.000339 | -5.741 | 1.29E-08 |  |
|  |  | Health | 0.000562 | 0.000366 | 1.535 | 0.12515 |  |
|  |  | Education | 4.92E-05 | 0.000193 | 0.256 | 0.79827 |  |
|  |  | Services | -0.0003 | 0.000108 | -2.778 | 0.00558 |  |
|  |  | Living | 6.86E-05 | 7.67E-05 | 0.895 | 0.37102 |  |
|  |  |  |  |  |  |  |  |
|  | LM | (Intercept) | 0.7685 | 0.1433 | 5.365 | 1.04E-07 | 0.2477 |
|  |  | Global Balance (Sn/Sb) | 0.08382 | 0.04174 | 2.008 | 0.0449 |  |
|  |  | Balance 2 (Co/Ni) | 0.233 | 0.1379 | 1.69 | 0.0915 |  |
|  |  | Income | 0.000329 | 0.000135 | 2.445 | 0.0147 |  |
|  |  | Employment | -0.002 | 0.00034 | -5.87 | 6.16E-09 |  |
|  |  | Health | 0.000603 | 0.000367 | 1.643 | 0.1007 |  |
|  |  | Education | 9.21E-05 | 0.000194 | 0.475 | 0.6351 |  |
|  |  | Services | -0.00028 | 0.000108 | -2.603 | 0.0094 |  |
|  |  | Living | 9.05E-05 | 7.77E-05 | 1.165 | 0.2442 |  |

Table 2: Summary of linear (LM) and generalised linear model (GLM) regression results for Standardised Incidence Rates (SIRs) for Chronic Kidney Disease (CKD) for all ages >16 yrs with balances identified by *selbal* and Multiple Deprivation domains of income, employment, health, education, services and living.

| Dependent variable | Model |  | Estimate | Std. Error | t value | Pr(>\|t\|) | R-Squared |
| --- | --- | --- | --- | --- | --- | --- | --- |
| lnSIR CKDu | GLM | (Intercept) | 0.9392 | 0.1759 | 5.34 | 1.73E-07 |  |
|  |  | Income | 0.00005826 | 0.000188 | 0.31 | 0.75699 |  |
|  |  | Employment | -0.0006196 | 0.000445 | -1.391 | 0.16512 |  |
|  |  | Health | 0.0003899 | 0.000479 | 0.813 | 0.41669 |  |
|  |  | Education | 0.000004968 | 0.000233 | 0.021 | 0.98302 |  |
|  |  | Services | 0.000136 | 0.000141 | 0.965 | 0.33542 |  |
|  |  | Living | -0.0002682 | 0.0001 | -2.671 | 0.00794 |  |
|  |  | Global Balance (Cr/Ni) | 0.5152 | 0.1726 | 2.984 | 0.00305 |  |
|  |  |  |  |  |  |  |  |
|  | LM | (Intercept) | 0.9392 | 0.1759 | 5.34 | 1.73E-07 | 0.0773 |
|  |  | Global Balance (Cr/Ni) | 0.5152 | 0.1726 | 2.984 | 0.00305 |  |
|  |  | Income | 0.00005826 | 0.000188 | 0.31 | 0.75699 |  |
|  |  | Employment | -0.0006196 | 0.000445 | -1.391 | 0.16512 |  |
|  |  | Health | 0.0003899 | 0.000479 | 0.813 | 0.41669 |  |
|  |  | Education | 0.000004968 | 0.000233 | 0.021 | 0.98302 |  |
|  |  | Services | 0.000136 | 0.000141 | 0.965 | 0.33542 |  |
|  |  | Living | -0.0002682 | 0.0001 | -2.671 | 0.00794 |  |
|  |  |  |  |  |  |  |  |
|  | LM | (Intercept) | 0.8351 | 0.1888 | 4.423 | 1.32E-05 | 0.0835 |
|  |  | Global Balance (Cr/Ni) | 0.4076 | 0.1867 | 2.183 | 0.0297 |  |
|  |  | Balance 2 As/Mo | 0.1233 | 0.08237 | 1.497 | 0.1354 |  |
|  |  | Income | 0.00004206 | 0.000188 | 0.224 | 0.8232 |  |
|  |  | Employment | -0.0005227 | 0.000449 | -1.164 | 0.2454 |  |
|  |  | Health | 0.0003179 | 0.000481 | 0.661 | 0.5091 |  |
|  |  | Education | -3.193E-07 | 0.000233 | -0.001 | 0.9989 |  |
|  |  | Services | 0.0001274 | 0.000141 | 0.905 | 0.3663 |  |
|  |  | Living | -0.0002608 | 0.0001 | -2.598 | 0.0098 |  |

Table 3: Summary of linear (LM) and generalised linear model (GLM) regression results for Standardised Incidence Rates (SIRs) for Chronic Kidney Disease of uncertain aetiology (CKDu) with balances identified by *selbal* and Multiple Deprivation domains of income, employment, health, education, services and living.

| Dependent variable | Model |  | Estimate | Std. Error | t value | Pr(>\|t\|) |
| --- | --- | --- | --- | --- | --- | --- |
| CKDu | Tweedie (link.power = 0) | Intercept | 0.96143 | 0.21186 | 4.538 | 7.92E-06 |
|  |  | Cr/Ni | 0.3328 | 0.13376 | 2.488 | 0.0133 |
|  |  | As/Mo | 0.11765 | 0.05237 | 2.247 | 0.0253 |
|  |  | Co/Ni | 0.2367 | 0.13552 | 1.747 | 0.0816 |

Table 4: Summary of Tweedie model regression results for Standardised Incidence Rates (SIRs) for Chronic Kidney Disease of uncertain aetiology (CKDu) using *selbal* balance approach

| Dependent variable | Model |  | Estimate | Std. Error | t value | Pr(>\|t\|) |  |
| --- | --- | --- | --- | --- | --- | --- | --- |
| CKDu | Tweedie (link.power = 1) | Intercept | 0.92455 | 0.070685 | 13.08 | <2e-16 |  |
|  |  | x.ilr1 | -0.030789 | 0.019271 | -1.598 | 0.11043 | Co/As |
|  |  | x.ilr2 | -0.00616 | 0.019533 | -0.315 | 0.75254 | Cr/As,Co |
|  |  | x.ilr3 | 0.03206 | 0.017231 | 1.861 | 0.06309 | Cu/As,Co,Cr |
|  |  | x.ilr4 | 0.038971 | 0.012915 | 3.018 | 0.00261 | Mo/As,Cr,Co |
|  |  | x.ilr5 | 0.063606 | 0.02662 | 2.389 | 0.01706 | Ni/As,Co,Cr,Mo |
|  |  | x.ilr6 | -0.009005 | 0.009438 | -0.954 | 0.34026 | Pb/As,Co,Cr,Mo,Ni |
|  |  | x.ilr7 | 0.001786 | 0.009534 | 0.187 | 0.85147 | Sb/As,Co,Cr,Mo,Ni,Pb |
|  |  | x.ilr8 | 0.016573 | 0.007809 | 2.122 | 0.03406 | Sn/As,Co,Cr,Mo,Ni,Pb,Sb |
|  |  | x.ilr9 | 0.053119 | 0.031548 | 1.684 | 0.09254 | V/As,Co,Cr,Mo,Ni,Pb,Sb,Sn |
|  |  | x.ilr10 | 0.036338 | 0.013842 | 2.625 | 0.00879 | Zn/As,Co,Cr,Mo,Ni,Pb,Sb,Sn,V |
|  |  |  |  |  |  |  |  |
|  |  |  |  |  |  |  |  |
|  | Tweedie (link.power = 0) | Intercept | 1.02322 | 0.03106 | 32.943 | <2e-16 |  |
| CKDu |  | Cr/Ni | -0.02185 | 0.017814 | -1.227 | 0.2203 |  |
|  |  | As/Mo | -0.014606 | 0.007342 | -1.989 | 0.0469 |  |
|  |  | Co/Ni | -0.01492 | 0.017568 | -0.849 | 0.3959 |  |

Table 5: Summary of Tweedie regression results for Standardised Incidence Rates (SIRs) for Chronic Kidney Disease of uncertain aetiology (CKDu) using *compositions* ilr base approach


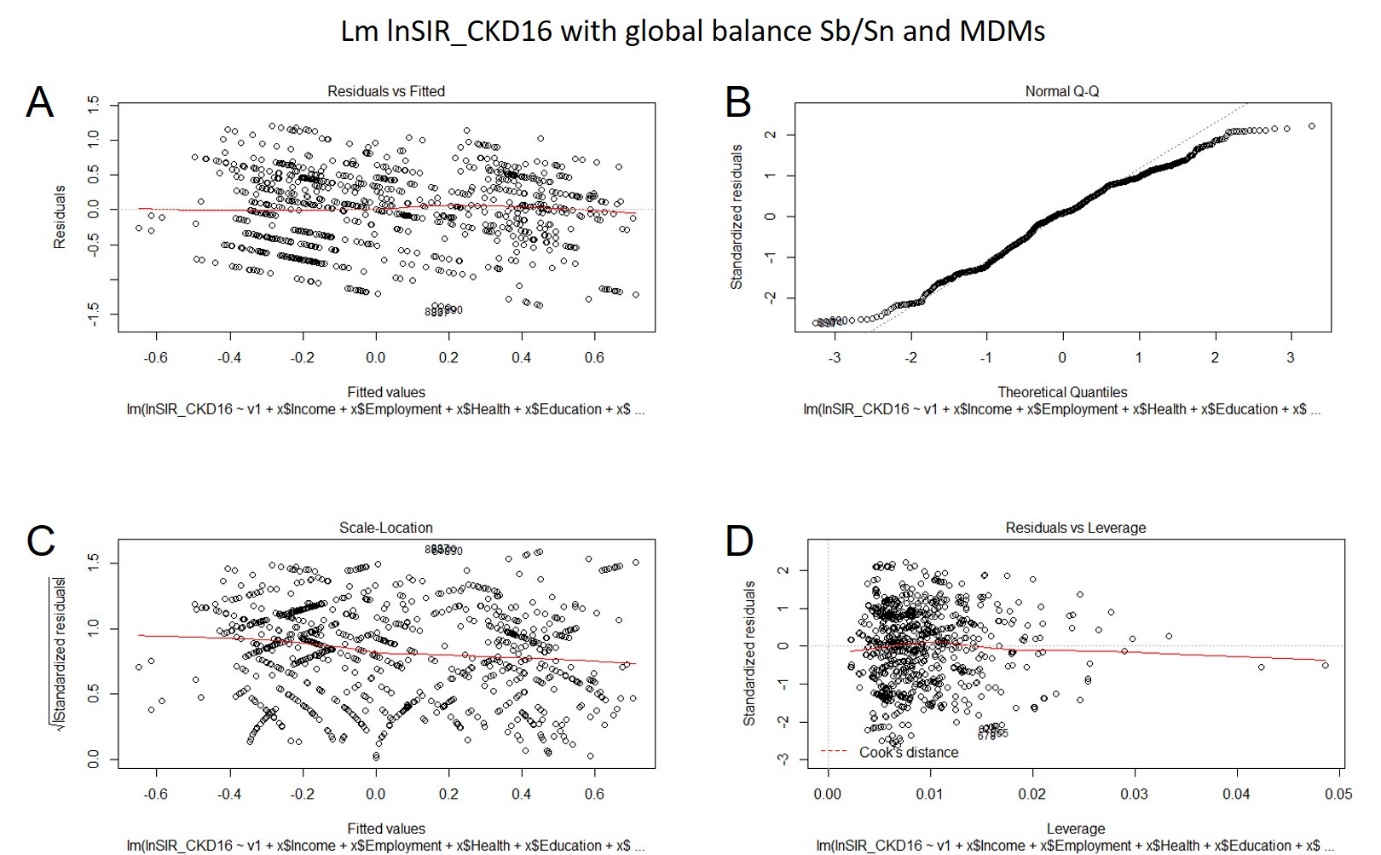


Figure 1: Linear model regression (LM) summary for log(SIR_CKD) >16 years with global balance Sb/Sn and Multiple Deprivation Indices (MDMs) A) Residual vs Fitted; B) Normal Q-Q plot; C) Scale Location and D) Residuals vs Leverage.


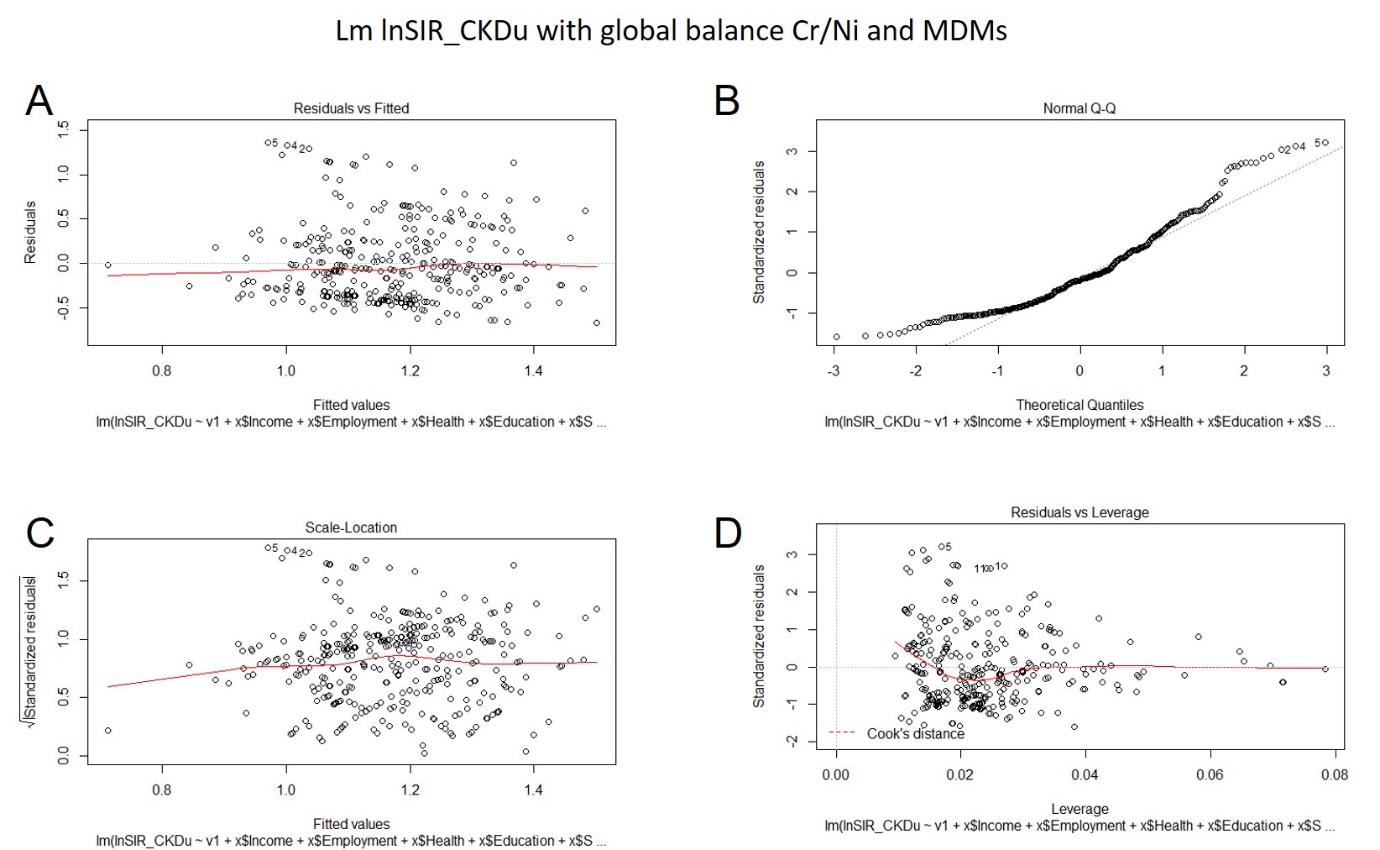


Figure 2: Linear model regression (LM) summary for log(SIR_CKDu) with global balance Cr/Ni and Multiple Deprivation Indices (MDMs) A) Residual vs Fitted; B) Normal Q-Q plot; C) Scale Location and D) Residuals vs Leverage.


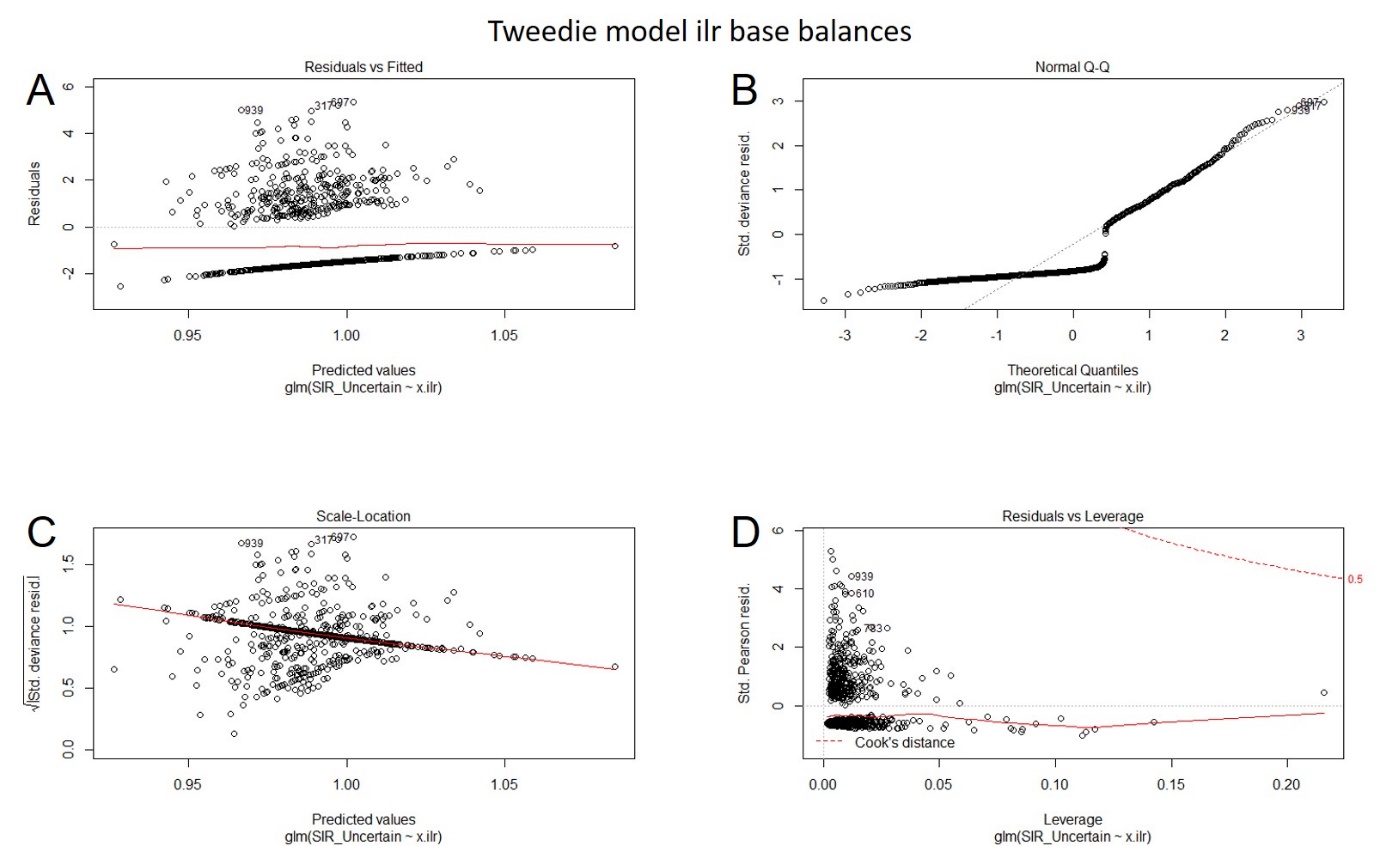


Figure 3: Tweedie model regression summary for log(SIR_CKDu) with ilr base balances and Multiple Deprivation Indices (MDMs) A) Residual vs Fitted; B) Normal Q-Q plot; C) Scale Location and D) Residuals vs Leverage.


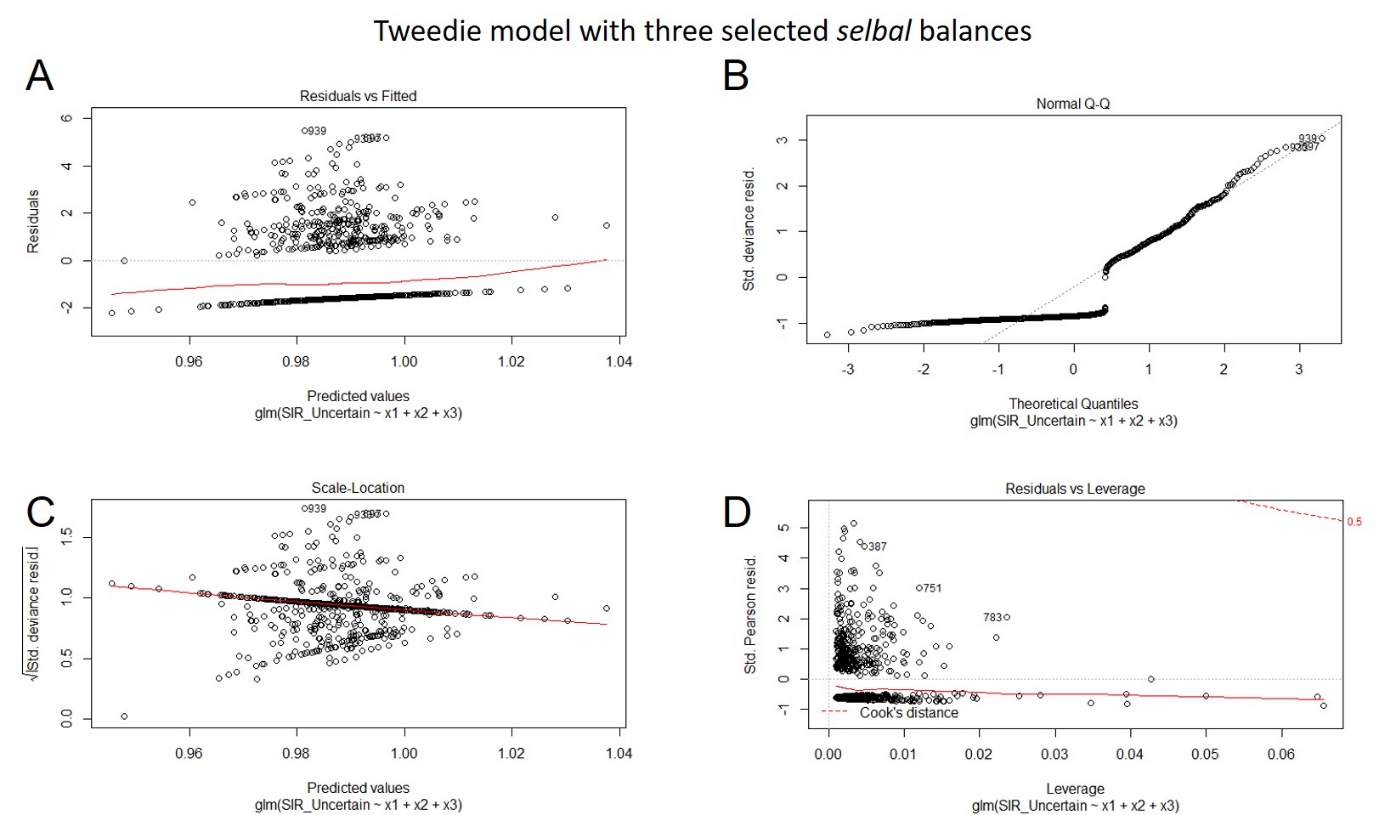


Figure 4: Tweedie model regression summary for log(SIR_CKDu) with selected balances Sb/Sn, As/Mo and Co/Ni from *selbal* and Multiple Deprivation Indices (MDMs) A) Residual vs Fitted; B) Normal Q-Q plot; C) Scale Location and D) Residuals vs Leverage.
